# Antihyperglycaemia and related gene expressions of aqueous extract of *Gongronema latifolium* leaf in alloxan-induced diabetic rats

**DOI:** 10.1080/13880209.2019.1657907

**Published:** 2019-09-12

**Authors:** Basiru O. Ajiboye, Babatunji E. Oyinloye, Precious E. Agboinghale, Sunday A. Onikanni, Emeka Asogwa, Abidemi P. Kappo

**Affiliations:** a Phytomedicine and Nutraceutical Research Laboratory, Department of Biochemistry, College of Sciences, Afe Babalola University, Ado-Ekiti, Nigeria;; b Biotechnology and Structural Biology (BSB) Group, Department of Biochemistry and Microbiology, University of Zululand, KwaDlangezwa, South Africa;; c Central Research and Diagnostic Laboratory, Ilorin, Nigeria

**Keywords:** Diabetes mellitus, ethnobotanical doses, intraperitoneal, hexokinase*;* GLUT-2, GLUT-4, lipid peroxidation

## Abstract

**Context:** *Gongronema latifolium* Benth (Asclepiadaceae) has been highly utilized in controlling diabetes mellitus traditionally in the eastern part of Nigeria.

**Objectives:** Antihyperglycaemic and related gene expressions of aqueous extract of *Gongronema latifolium* leaf in alloxan-induced diabetic rats.

**Materials and methods:** Forty-eight female Wistar rats were induced intraperitoneally using alloxan (150 mg/kg body weight). The rats were separated into six groups (*n* = 8) as follows: non-diabetic control, diabetic control, diabetic rats administered 5 mg/kg body weight of metformin, and diabetic rats administered 6.36, 12.72 and 25.44 mg/kg body weight (ethnobotanical doses) of *G. latifolium* orally daily. On the 14th day, the animals were sacrificed and different antihyperglycaemic parameters were evaluated as well as its related gene expressions.

**Results:** Diabetic rats administered three doses of aqueous extract of *G. latifolium* significantly (*p* < 0.05) lowered the fasting blood glucose, glycated haemoglobin, serum lipid profiles, lipid peroxidation (5.62–1.2 μ/mg protein) levels, as well as gene expression of glucose-6-phosphatase in alloxan-induced diabetic rats. There was a significant (*p* < 0.05) increase in the liver glycogen content (16.23–112.5 mg glucose/2 g), antioxidant enzymes activities, glucose transporter (GLUT-2 and GLUT-4) levels and relative gene expression of hexokinase in diabetic rats administered different doses of aqueous extract of *G. latifolium*.

**Discussion and conclusions:** It can be deduced that the aqueous extract of *G. latifolium* leaf at these doses may be useful in managing diabetes mellitus and its associated complications. Therefore, this extract may be a potent antidiabetic agent in clinical therapy in the future.

## Introduction

Diabetes mellitus, often called diabetes, is a group of metabolic syndromes known with a high concentration of fasting blood glucose over a persistent period of time (Hall 2015). Diabetes is a very complex disease connected to impairment, malfunction and failure of diverse organs notably the kidneys, eyes, nerves, heart, blood vessels, etc. (American Diabetes Association [ADA 2009]). Some of the symptoms of diabetes mellitus include excessive thirst, muscle wasting, blurred vision, polyuria, and sometimes insatiable appetite. Approximately more than 4 million deaths are recorded globally from diabetes mellitus yearly (International Diabetes Federation [IDF 2017]).

Currently, the use of modern drugs for the management of diabetes mellitus has been associated with a series of negative effects, which is a concern for researchers globally. Also, the uses of modern drugs have been constrained by their pharmacokinetic properties, secondary failure rates, and accompanying negativity (Stalin et al. 2013). Thus, the search for a new compound with no or limited side effects is very crucial in the management of diabetes. Different medicinal plants have been recognized in Africa and are used as herbs, mainly in local communities by traditional medicine (Denton et al. 2004). Some of these therapeutic herbs are useful in the management of diabetes mellitus universally. In Nigeria, a series of plants have been documented in the treatment/management of many ailments, especially diabetes mellitus. These plants are frequently used in underdeveloped countries where many people do not have access to modern antidiabetic drugs (Abubakar et al. 2017). *Gongronema latifolium* Benth (Asclepiadaceae) is an example of an herb/plant believed to be helpful in managing diabetes mellitus and its complications.

*Gongronema latifolium,* commonly called “*utazi*” among the Igbo and “*arokeke*” in Yoruba speaking communities in Nigeria, is a native of south eastern Nigeria (Morebise et al. 2002). Ugochukwu et al. (2003) documented in Africa (especially in Nigeria) ethno-medicine that this plant has been helpful since ancient times in controlling diabetes mellitus. There is some evidence of the normoglycaemic, hypolipidaemic and antioxidative activity of the plant (Ugwu et al. 2010), but not on the uses of this plant based on ethnobotanical survey dosage in managing of diabetes mellitus. For drug discovery, it is imperative to use this medicinal plant the way it is being used locally to serve as scientific evidence for used doses (local dosage). Therefore, this study is designed to assess antihyperglycaemia and related gene expression of aqueous extract of *G. latifolium* leaf in alloxan-induced diabetic rats.

## Materials and methods

### Plant materials and authentication

*Gongronema latifolium* leaf was acquired in January 2019 at Ekpoma market, Ekpoma, Edo State, Nigeria. It was recognized and authenticated by Mr Odewo, a senior taxonomist at Forestry Research Institute of Nigeria (FRIN), Ibadan, Oyo State, Nigeria, with Herbarium number FHI: 112032.

### Chemicals and reagents

All the chemicals and reagents used in this experiment were secured from Sigma-Aldrich Inc. (St. Louis, MO, USA), while all enzymes assay kits were procured from Randox Laboratories Ltd., Antrim, UK.

### Processing of *G. latifolium* leaf

*Gongronema latifolium* leaf was dried at 25 °C for 30 d. Then pulverized into powder by an electric blender. The powder sample (200 g) was soaked in 2000 mL of distilled H_2_O for a day, filtered, and freeze-dried to obtain the dried extract. A cup (based on ethnobotanical survey) of the filtrate (250 mL) was freeze-dried and the yield obtained was equivalent to the intake of a 70 kg man. This was then extrapolated to get 12.72 mg/kg body weight (equivalent dose of 70 kg man), then multiply by 2 and divided by 2 to get high and low ethnobotanical doses, respectively; 25.44 and 6.36 mg/kg body weight according to Yakubu et al. (2014). The extract was weighed in a universal bottle using a weighing balance. The universal bottle containing the weighed extract was closed and kept at 4 °C for various studies.

### Experimental animals

Forty-eight (48) female Wistar rats of 120–135 g, acquired from the Animal Holding unit of Afe Babalola University, Ado-Ekiti, Nigeria. The rats were placed on drinkable water, food and room temperature at 25 °C. This study was approved by ABUAD Animal Ethical Committee (approval number 19/ABUADSCI/016).

### Animal grouping

The animals were divided into six groups (*n* = 8) as follows:

Group 1: non-diabetic control rats received distilled water (non-diabetic control)
Group 2: rat induced with diabetes but no treatment (diabetic control)
Group 3: diabetic rats administered 5 mg/kg body weight of metformin
Group 4: diabetic rats administered 6.36 mg/kg body weight of aqueous extract *G. latifolium* leaf
Group 5: diabetic rats administered 12.72 mg/kg body weight of aqueous extract *G. latifolium* leaf
Group 6: diabetic rats administered 25.44 mg/kg body weight of aqueous extract *G. latifolium* leaf.

### Diabetes induction

Fasting blood glucose levels of each rat were assessed subject to overnight fasting. Then 5 g of alloxan monohydrate was liquefied in 0.9% saline solution, which was used to induce diabetes mellitus into the rats (by single intraperitoneal injection) at a dose of 150 mg/kg body weight. Forty-eight hours after the induction, fasting blood glucose levels were evaluated via ACCU check glucometer. Rats whose fasting blood glucose levels were greater than or equal to 250 mg/dL were used (Ajiboye et al. 2018).

### Collection and processing of samples

The rats were sacrificed on the 14th day of administration using cervical dislocation. Then blood was collected from rats for serum analysis. Then allowed to stand at 25 °C for 1800 s (clotting process), centrifuged at 3000 *g* for 600 s and collected the supernatant. The excised liver was rinsed with buffer, homogenized with 0.1 M phosphate buffer (pH 6.4) and centrifuged at 4000 *g* for 15 min. Thereafter, both the serum and the obtained homogenate supernatant were properly labelled and kept for different analyses.

### Biochemical parameters determined

#### Estimation of fasting blood glucose

This was carried out via an Accu-chek advantage clinical glucometer (Ahmad et al. 2007).

#### Serum biochemical parameters determination

Glycated haemoglobin, insulin and lipid profiles were determined according to the procedure outlined in their assay kits, while enzyme-linked immunosorbant assay (ELISA) was used in insulin estimation (Islam et al. 2015).

#### Liver glycogen determination

This was carried out using the procedure outlined by Murat and Serfaty (1975).

#### Determination of oxidative stress biomarkers

Lipid peroxidation measured as malondialdehyde (MDA), superoxide dismutase (SOD), catalase (CAT) and glutathione peroxidase (GPx) existed was evaluated using the procedures outlined in their respective assay kits.

### Gene expression analysis of some carbohydrate metabolizing enzymes

The RNA was isolated from each rat liver using TRIzol Reagent (Thermo Fisher Scientific, Waltham, MA), then, removing DNA contaminant using DNAse I treatment (Thermo Fisher Scientific, Waltham, MA) by following manufacturer’s protocol. The obtained RNA was then quantified with the aid of Hitachi-U1900 spectrophotometer (Hitachi, Tokyo, Japan) at 260 nm. ProtoScript First Standard cDNA Synthesis Kit (NEB) was used in converting purified DNA-free RNA into cDNA immediately. Also, OneTaq^®^ 2X Master Mix (NEB) was used in PCR amplification using the following primer set:

Hexokinase

Forward: GTGTACAAGCTGCACCCGA
Reverse: CAGCATGCAAGCCTTCTTG

Glucose-6-phosphatase

Forward: GCTCCGTGCCTCTGATAAA
Reverse: CCACGAAAGATAGCGAGAGTAG

The expression level of the genes studied was normalized by GADPH, and the band density was measured using ImageJ is plotted as a bar graph as illustrated by Elekofehinti et al. (2018).

### Data analysis

Data were reported as the mean of eight replicates ± standard deviation (SD). Graph pad prism 5 (GPP) software (Graphpad Inc., La Jolla, CA) was used for all acquired results. One-way ANOVA was employed and *post hoc* was done using Tukey. A significant difference was obtained at *p* ˂ 0.05.

## Results

### Body weight of diabetic rats administered aqueous extract of *G. latifolium* leaf

**Figure 1.**
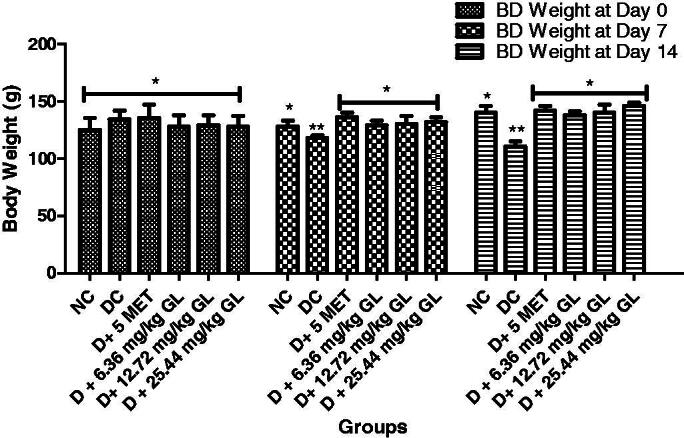
Aqueous leaf extract of *G. latifolium* on body weight of alloxan-induced diabetic rats. Values are expressed as mean ± standard deviation (SD) of eight replicates. Bar with the same * are not significantly different at *p* > 0.05. Bar with different * are significantly different at *p* < 0.05. NC: non-diabetic control; DC: diabetic control; D: diabetic; MET: metformin; GL: *Gongronema latifolium* leaf; BD: body weight.

At the start of the experiment, the rats body weights were not significant (*p* > 0.05) different from one another. However, at days 7 and 14, there was a significant (*p* < 0.05) reduction in the body weight of diabetic control rats compared with other groups. Whereas there were significant (*p* < 0.05) improvement in body weight of diabetic rats administered different doses of aqueous extract of *G. latifolium* leaf with no significant (*p* > 0.05) different with non-diabetic control rats at days 7 and 14 (Figure 1).

### Fasting blood glucose level of diabetic rats administered aqueous extract of *G. latifolium* leaf

**Figure 2.**
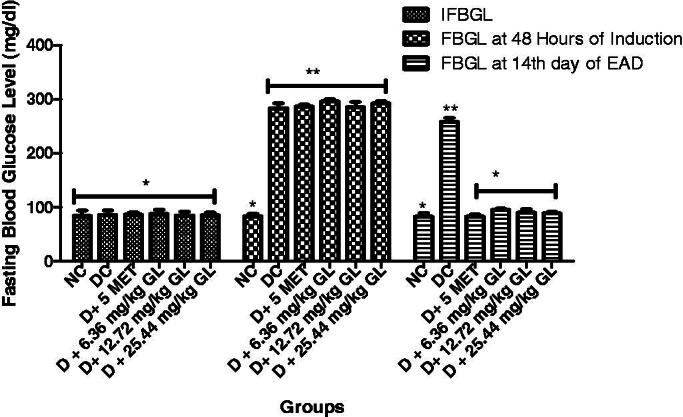
Aqueous leaf extract of *G. latifolium* on fasting blood glucose levels in alloxan-induced diabetic rats. Values are expressed as mean ± standard deviation (SD) of eight replicates. Bar with the same * are not significantly different at *p* > 0.05. Bar with different * are significantly different at *p* < 0.05. NC: non-diabetic control; DC: diabetic control; D: diabetic; MET: metformin; GL: *Gongronema latifolium* leaf; EAD: end of administration; FBGL: fasting blood glucose.

The result shows that the fasting blood glucose levels before induction were not significant (*p* > 0.05) different from each other. There was a significant (*p* < 0.05) increase in the fasting blood glucose level at 48 h of alloxan induction in all the groups except in the normal control. At the 14th day of administration of the three doses of aqueous extract of *G. latifolium* leaf, there was a significant (*p* < 0.05) reduction in the fasting blood glucose levels with no significant (*p* > 0.05) different from the non-diabetic control rats (Figure 2).

### Serum insulin of diabetic rats administered aqueous extract of *G. latifolium* leaf

**Figure 3.**
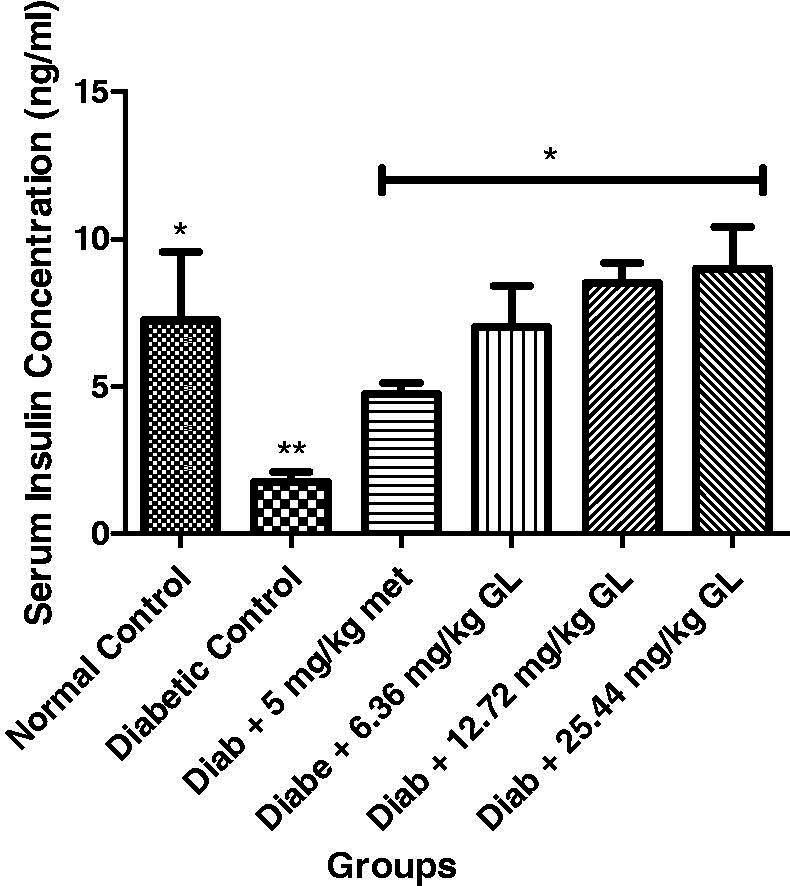
Serum insulin level of alloxan-induced diabetic rats after administration of *G. latifolium* leaf. Values are expressed as mean ± standard deviation (SD) of eight replicates. Bar with the same * are not significantly different at *p* > 0.05. Bar with different * are significantly different at *p* < 0.05. Normal control: non-diabetic control; GL: *Gongronema latifolium* leaf; Diab: diabetic; met: metformin.

There was a significant (*p* < 0.05) decrease in the serum insulin levels in diabetic control rats when compared with other groups. However, there was a significant (*p* < 0.05) increase in serum insulin levels in diabetic rats administered different doses aqueous extract of *G. latifolium* leaf and exhibited no significance (*p* > 0.05) different with non-diabetic control rats (Figure 3).

### Serum lipid profiles of diabetic rats administered aqueous extract of *G. latifolium* leaf

**Figure 4.**
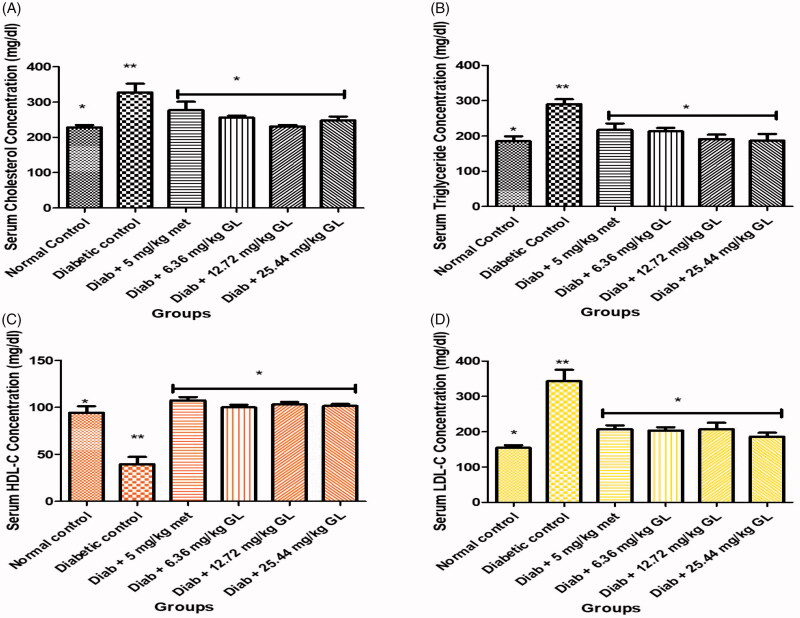
Serum lipid profile level of alloxan-induced diabetic rat after administration of *G. latifolium* leaf. Values are expressed as mean ± standard deviation (SD) of eight replicates. Bar with the same * are not significantly different at *p* > 0.05. Bar with different * are significantly different at *p* < 0.05. Normal control: non-diabetic control; GL: *Gongronema latifolium* leaf: met: metformin; Diab: diabetic; HDL-C: high-density lipoprotein cholesterol; LDL-C: low-density lipoprotein cholesterol.

The result showed that there was a significant (*p* < 0.05) increase in the levels of cholesterol, triglyceride and low-density lipoprotein (LDL) with significant (*p* < 0.05) reduction in high-density lipoprotein (HDL) in the diabetic control rats when compared with other groups. At the end of the experiment, oral administration of three doses of aqueous extract of *G. latifolium* leaf shows a significant (*p* < 0.05) reduction in the levels of cholesterol, triglycerides and LDL with a significant (*p* < 0.05) increase in HDL and compared favourably with the non-diabetic control group (Figure 4).

### Glycated haemoglobin of diabetic rats administered aqueous extract of *G. latifolium* leaf

**Figure 5.**
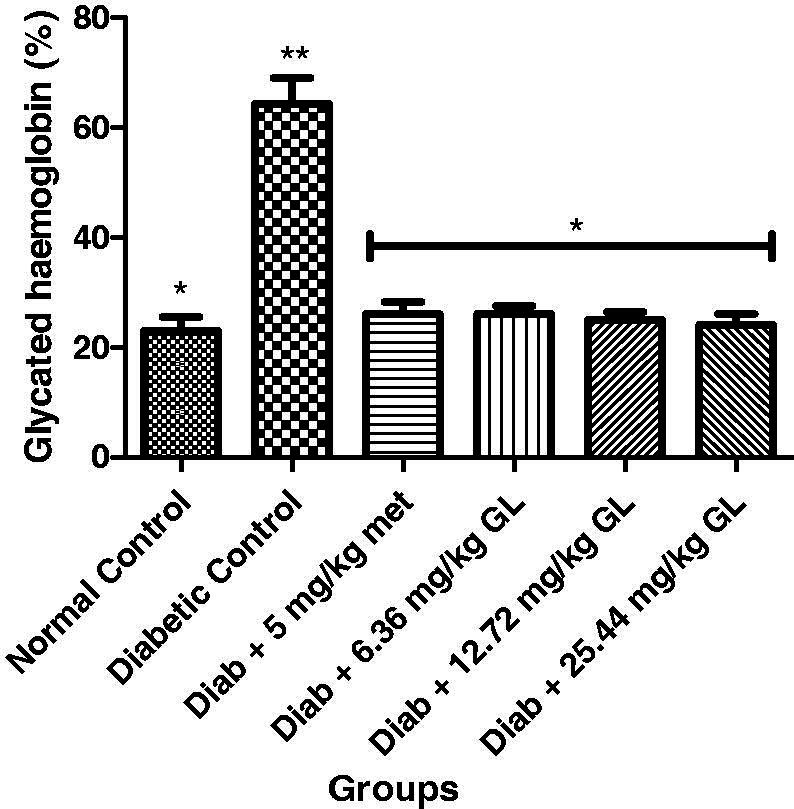
Glycated haemoglobin level in alloxan-induced diabetic rats after administration of aqueous extract of *G. latifolium* leaf. Values are expressed as mean ± standard deviation (SD) of eight replicates. Bar with the same * are not significantly different at *p* > 0.05. Bar with different * are significantly different at *p* < 0.05. Normal control: non-diabetic control; met: metformin; Diab: diabetic; GL: *Gongronema latifolium* leaf.

Glycated haemoglobin level was significantly (*p* < 0.05) decreased in the diabetic control group compared with other groups. But at the 14th day of administration of different doses of aqueous extract of *G. latifolium* leaf, there was a significant (*p* < 0.05) decrease in the levels of glycated haemoglobin and compared favourably with both the metformin and non-diabetic control groups (Figure 5).

### Liver glycogen of diabetic rats administered aqueous extract of *G. latifolium* leaf

**Figure 6.**
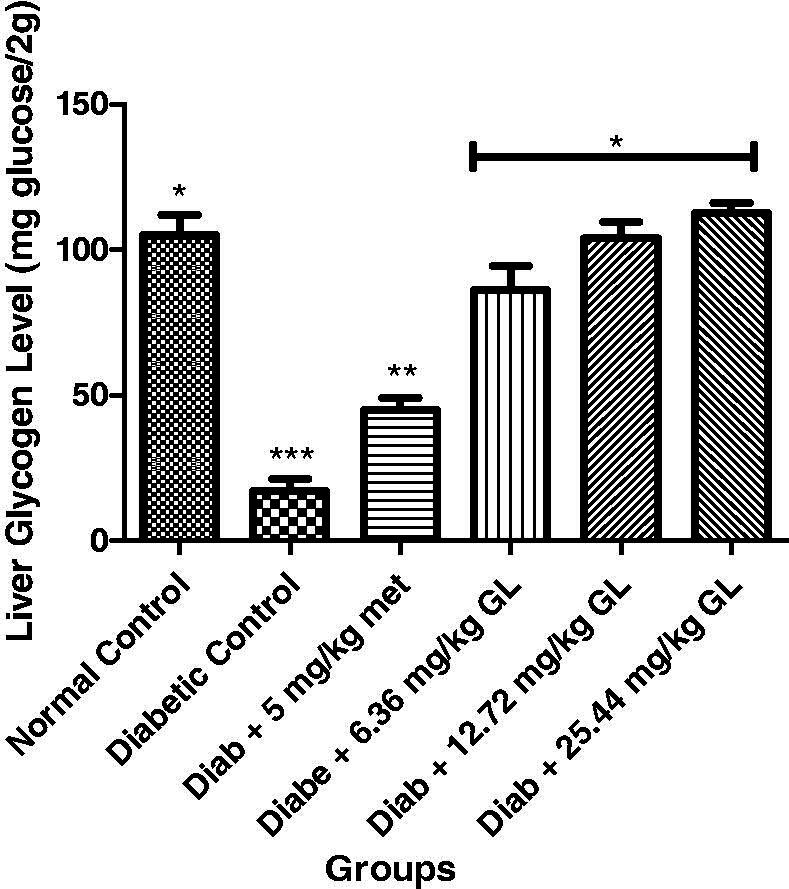
Liver glycogen level in alloxan-induced diabetic rats after administration of aqueous extract of *G. latifolium* leaf. Values are expressed as mean ± standard deviation (SD) of eight replicates. Bar with the same * are not significantly different at *p* > 0.05. Bar with different * are significantly different at *p* < 0.05. Normal control: non-diabetic control; met: metformin; diab: diabetic; GL: *Gongronema latifolium* leaf.

There was a significant (*p* < 0.05) decrease in liver glycogen of diabetic control rat compared to other groups. But at the end of the experiment, there was a significant (*p* < 0.05) increase in liver glycogen levels in diabetic rats administered three different doses of aqueous extract of *G. latifolium* leaf (Figure 6).

### Oxidative stress biomarkers of diabetic rats administered aqueous extract of *G. latifolium* leaf

**Figure 7.**
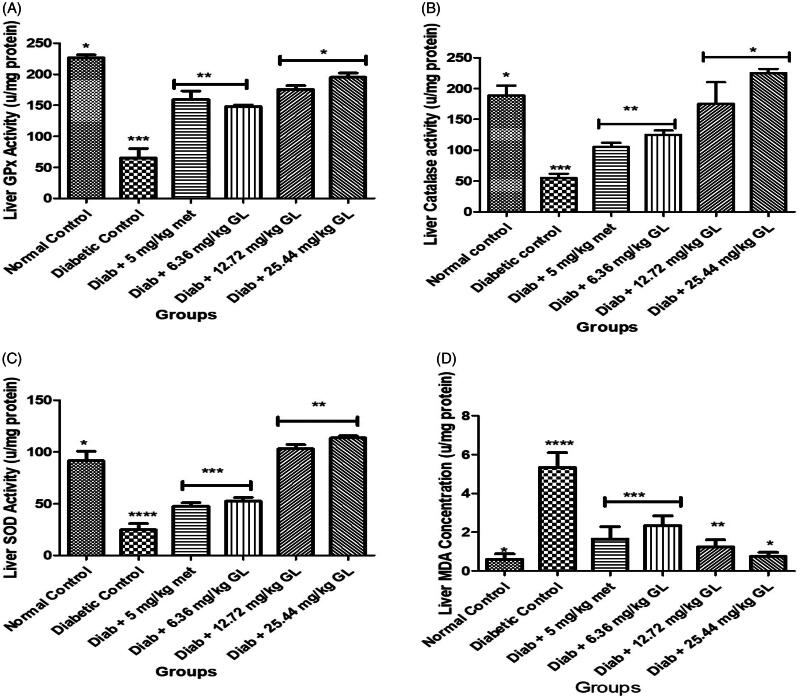
Antioxidant enzyme activities and lipid peroxidation level after administration of aqueous extract of *G. latifolium* leaf. Values are expressed as mean ± standard deviation (SD) of eight replicates. Bar with the same * are not significantly different at *p* > 0.05. Bar with different * are significantly different at *p* < 0.05. Normal control: non-diabetic control; met: metformin; diab: diabetic; GL: *Gongronema latifolium* leaf; GPx: gluthathione peroxidase; SOD: superoxidase dismutase; MDA: malondialdehyde; CAT: catalase.

There was a significant (*p* < 0.05) decrease in CAT, SOD and GPx activities with a significant (*p* < 0.05) increase in MDA levels in diabetic control rat compared to other groups. However, there was a significant (*p* < 0.05) increase in the activities of CAT, SOD and GPx with a significant (*p* < 0.05) reduction in MDA levels in diabetic rats administered different doses of aqueous extract of *G. latifolium* leaf and compared favourably with non-diabetic control (Figure 7).

### Hexokinase and glucose-6-phosphatase gene expression of diabetic rats administered aqueous extract of *G. latifolium* leaf

**Figure 8.**
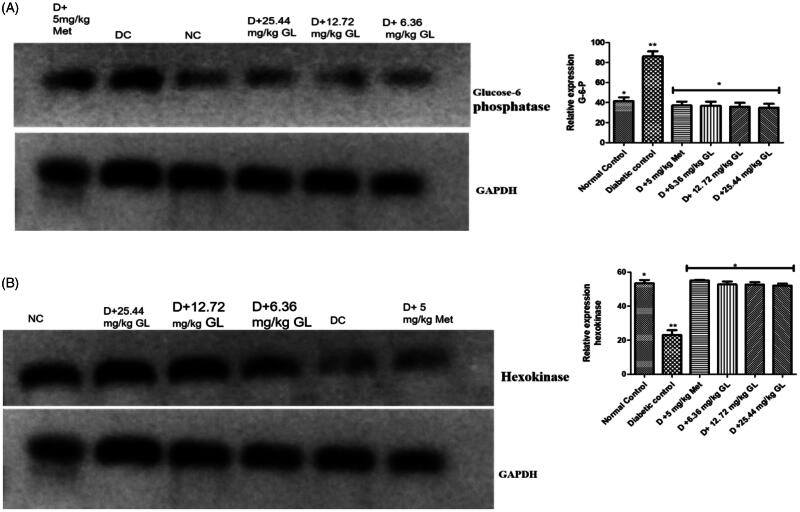
(a) Hepatic hexokinase gene expression in alloxan-induced diabetic rat after administration of aqueous extract of *G. latifolium* leaf. (b) Hepatic glucose-6-phosphatase gene expression in alloxan-induced diabetic rats after administration of aqueous extract of *G. latifolium* leaf. Values are expressed as mean ± standard deviation (SD) of eight replicates. Bar with the same * are not significantly different at *p* > 0.05. Bar with different * are significantly different at *p* < 0.05. NC or normal control: non-diabetic control; DC: diabetic control; D: diabetic; met: metformin; GL: *Gongronema latifolium* leaf.

The result showed that there was a significant (*p* < 0.05) reduction in gene expression of hexokinase with a significant (*p* < 0.05) increase in gene expression of glucose-6-phosphatase in liver of diabetic control rats when compared with other groups. Conversely, there was a significant (*p* < 0.05) increase in gene expression of hexokinase with a significant (*p* < 0.05) decrease in gene expression of glucose-6-phosphatase in liver of diabetic rats administered three different doses of aqueous extract of *G. latifolium* leaf and diabetic rats administered metformin (Figure 8(a,b)).

### Glucose transporters (GLUT 2 and GLUT 4) of diabetic rats administered aqueous extract of *G. latifolium* leaf

**Figure 9.**
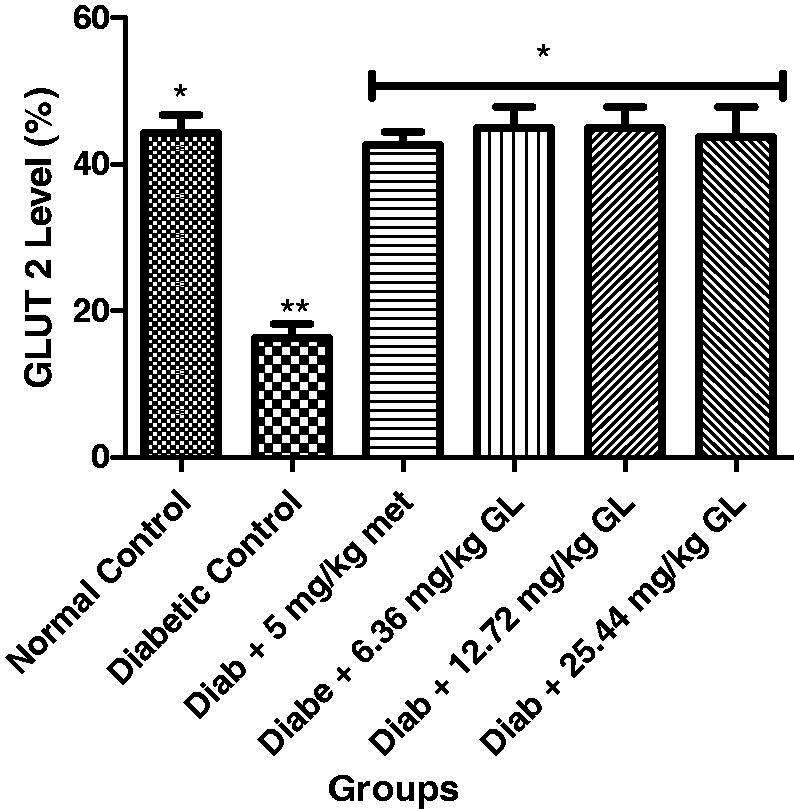
Glucose transporter (GLUT-2) level after administration aqueous extract of *G. latifolium* leaf. Values are expressed as mean ± standard deviation (SD) of eight replicates. Bar with the same * are not significantly different at *p* > 0.05. Bar with different * are significantly different at *p* < 0.05. Normal control: non-diabetic control; met: metformin; Diab: diabetic; GL: *Gongronema latifolium* leaf.

**Figure 10.**
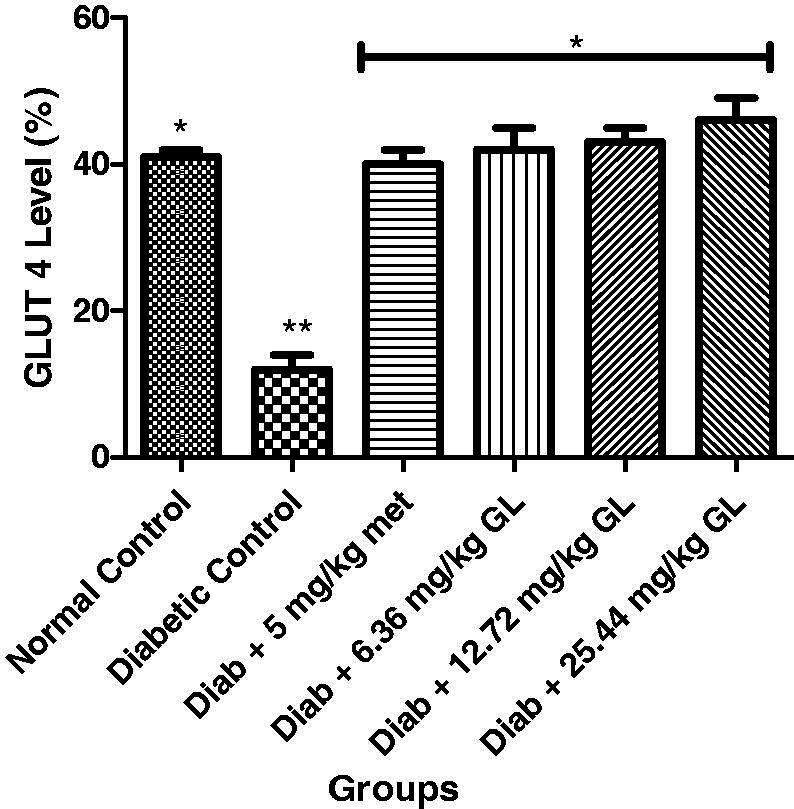
Glucose transporter (GLUT4) level after administration aqueous extract of *G. latifolium* leaf. Values are expressed as mean ± standard deviation (SD) of eight replicates. Bar with the same * are not significantly different at *p* > 0.05. Bar with different * are significantly different at *p* < 0.05. Normal control: non-diabetic control; met: metformin; Diab: diabetic; GL: *Gongronema latifolium* leaf.

As shown in Figures 9 and 10, there was a significant (*p* < 0.05) decrease in the levels of GLUT 2 and GLUT 4 in diabetic control when compared with other groups. But at the end of the experiment, there was a significant (*p* < 0.05) increase in the levels of GLUT 2 and GLUT 4 of diabetic animals administered three different doses of aqueous extract of *G. latifolium* leaf and they exhibited no significance (*p* > 0.05) different with the non-diabetic control group.

## Discussion

In this study, the aqueous extract of *G. latifolium* leaf surprisingly had the ability to enhance the body weight of induced diabetic rats. It has been documented by Adiga et al. (2010) amongst several researchers that alloxan-induced rats are characterized with body weight loss possibly owing to breaking down of proteins and the inability to provide amino acid for gluconeogenesis in insulin deficiency causing muscle wasting and breakdown of tissues in diabetic rats. But this was restored in diabetic rats administered aqueous extract of *G. latifolium* leaf probably due to the enhancement of insulin secretion.

Alloxan was used as the inducing agent to induce diabetes mellitus by prompting selective damage of the pancreatic β-cells producing insulin (Szkudelski 2001). Damage to the beta-pancreatic cells affects the release of insulin which results in a condition known as hyperglycaemia. The ability of the aqueous extract of *G. latifolium* leaf to reduce hyperglycaemia to normoglycaemia may be associated to its ability to inhibit the absorption of glucose from the intestine to aid glucose release from the liver or by enhancing the number of insulin receptors so as to improve the sensitivity of target tissues to insulin levels. This may be associated with the antioxidant nature of the *G. latifolium* leaf extract which perhaps regenerates the damaged beta cells.

Individuals suffering from diabetes mellitus have been linked with an increase in low-density lipoprotein cholesterol (LDL-c concentration, known as “bad cholesterol” with a decrease concentration of high-density lipoprotein cholesterol (HDL-c) called “good cholesterol”. Excessive free radical’s accumulation in the body system of diabetic mellitus patient has been known as a key factor responsible for the increase in cardiovascular diseases and hypertension rate (Wang et al. 2007). Probably, the antioxidant nature of the aqueous extract of *G. latifolium* leaf is perhaps responsible for the enhancement in the concentration of good cholesterol (HDL) and decreases in the levels of bad cholesterol. This may ultimately lead to a decrease in the rate of cardiovascular diseases and hypertension in diabetes mellitus individuals. The abnormal rise in the level of glycated haemoglobin (HbA1c) may be attributed to the effect of alloxan which induced hyperglycaemia and leads to pancreatic beta-cell destruction (Gandhi & Sasikumar 2012). Glycated haemoglobin is a measurement that reflects both fasting and postprandial glucose concentrations over a period of 2-weeks. Glycated haemoglobin measures the degree of control of glucose level for a prolonged time. Reductions in the values of glycated haemoglobin in the diabetic rats administered aqueous extract of *G. latifolium* leaf support its normoglycaemic effect.

Liver glycogen is an important bio-marker to assess the normoglycaemic effect of drugs or plant extracts. The increase in liver glycogen level of diabetic rats administered aqueous extract of *G. latifolium* leaf may be attributed to its tendency to suppress glycogen phosphorylase. A key enzyme in glycogenolysis that is activated by glucagon and epinephrine to release glucose to the blood, by suppressing the glycogen phosphorylase. Thus, preventing glucose from entering the blood and consequently reducing the glucose levels in large amounts (Ajiboye et al. 2018). Furthermore, an enhancement in liver glycogen noticed in diabetic rats administered aqueous extract of *G. latifolium* leaf may be linked to an increase in the insulin secretion, as discussed earlier.

The significant increase in the activities of SOD, CAT and GPx in diabetic rats administered aqueous extract of *G. latifolium* leaf shows that it has free radicals quenching ability. Diabetes mellitus is known to decrease antioxidant enzymes (especially SOD, CAT and GPx) activities in the body system of such patients. This may enhance organ damage linked to the accumulation of reactive oxygen species that probably trigger an increase in lipid peroxidation in diabetes mellitus condition (Pari & Latha 2005). Moreover, the antioxidant nature of the aqueous extract of *G. latifolium* leaf may be associated with a decrease in the lipid peroxidation level observed in the current study.

The decrease in hexokinase gene expression observed in diabetic rats administered aqueous extract of *G. latifolium* leaf may be as a result of the effortless ability of the extract to directly stimulate glycolysis, probably because of an increase in insulin secretion. Since hexokinase is the first regulating enzyme in intracellular glucose metabolism which catalyzes the phosphorylation of glucose to glucose-6-phosphate in the tissues by increased glucose removal. Interestingly, the potential of the extract to obstruct gluconeogenesis occurs in down regulation of glucose-6-phosphatase gene expression. Glucose-6-phosphatase is the last stage of gluconeogenesis pathway which catalyzes the breaking down of glucose-6-phosphate to glucose (Kazeem et al. 2013). The inhibition of this enzyme in diabetic rats administered aqueous extract of *G. latifolium* leaf may be due to increasing glycogen synthesis due to an increase in insulin secretion.

The observed increases in the levels of GLUT 2 and GLUT 4 in diabetic rats administered aqueous extract of *G. latifolium* leaf may be due to an increase in insulin secretion. While the decrease in these glucose transporters may be linked to a decrease in insulin secretion in diabetic mellitus state. These results agree with the earlier study by Al-Shaque et al. (2005).

## Conclusions

Taken together, results from this study show that aqueous extract of *G. latifolium* leaf has antidiabetic activities useful in managing diabetic complications. Also, to the best of our knowledge, this is the first research on the ethnobotanical investigation on the local benefit of this plant in the management of diabetes mellitus. In addition, this study also revealed that *G. latifolium* leaf had a modulatory effect on the level of hepatic hexokinase and glucose-6-phosphatase gene expression in a diabetic animal model.
